# Proximate Composition, Minerals and Antioxidant Activity of Artichoke Leaf Extracts

**DOI:** 10.1007/s12011-019-01806-3

**Published:** 2019-07-08

**Authors:** Wioletta Biel, Robert Witkowicz, Ewa Piątkowska, Cezary Podsiadło

**Affiliations:** 1 Department of Pig Breeding, Animal Nutrition and Food, West Pomeranian University of Technology, Szczecin, Poland; 2 Institute of Plant Production, University of Agriculture, Krakow, Poland; 3 Department of Human Nutrition, University of Agriculture, Krakow, Poland; 4 Department of Agronomy, West Pomeranian University of Technology, Szczecin, Poland

**Keywords:** *Cynara scolymus*, Leaf extracts, Element ratios, Total phenolic content, Antioxidant activity

## Abstract

In this study, leaf extracts from the Green Globe cultivar of artichoke (*Cynara scolymus* L.), a herbaceous plant of the Asteraceae family, were analyzed to determine the levels of basic nutrients, selected macroelements (K, P, Ca, Mg, and Na) and microelements (Zn, Fe, Mn, Cr, Pb, Cd, and Ni), and their ratios. The antioxidant activity (aa) of the extract was evaluated using ABTS˙^+^ and DPPH˙^+^ radicals and the ferric reducing antioxidant power assay (III) (FRAP). Total polyphenolic content was also determined. Macroelement concentrations in the artichoke leaf extract can be presented in descending order as follows: K > P > Ca > Mg > Na. Microelement content in the extract was as follows: Zn > Fe > Cr > Mn. We determined the ratios of elements in artichoke leaf extracts and compared them against the recommended dietary allowance, adequate intake, or tolerable upper intake level. Mean total phenolic content in artichoke leaf extracts was high – 2795 mg CAE/100 g dry matter (DM). The ABTS˙^+^ assay showed a very high ability of artichoke extract to scavenge free radicals (79.74%), and the antioxidant capacity measured at 1060.8 Trolox/1 g DM. The results show that artichoke extract is a valuable source of minerals and antioxidants that could have applications in the prevention of chronic non-communicable diseases caused by oxidative damage.

## Introduction

Free radicals and their ability to induce oxidative stress play an important role in the pathogenesis of chronic non-communicable diseases. In the search for substances supporting natural antioxidant defense, particular attention is paid to secondary metabolites derived from plants with recognized dietary or therapeutic effects. It is believed that the compounds with the strongest antioxidant properties are polyphenols: flavonoids, isoflavones, anthocyanins, and catechins. Weaker antioxidant properties are exhibited by carotenoids or vitamins C and E. As many of these compounds are not synthesized by the human body, intake through food is of great importance for human health, especially in the protection against free radicals.

One of the important natural sources of antioxidants is the globe artichoke (*Cynara scolymus* L.). The beneficial properties of this plant have been known since antiquity, and nowadays, it is used in medicine, herbiculture, food, feed, and many other industries [1–4]. In terms of nutrients and bioactive substances, the highest concentrations are recorded in its leaves*,* which are mainly used to produce extracts [5]. Artichoke extracts can be added to food, mainly meat, both for its aroma and to protect food from lipid and protein oxidation.

Interest in artichoke extract results from its multifaceted therapeutic effects. The most characteristic effect is the stimulation of bile secretion. Extracts exert a diastolic effect on the gallbladder and bile ducts, causing an increase in the production of bile acid by liver cells [6]. Many authors emphasize that long-term dietary supplementation with artichoke extract significantly improves blood lipid profile [2, 7–9]. Antioxidants contained in the extract can also protect the liver from the harmful effects of toxins, heavy metals, and other chemicals [5, 10].

Furthermore, artichoke extract has also shown a cholesterol-reducing effect. A study showed the beneficial effect of artichoke leaf extract on serum and hepatic lipid levels and hepatic and cardiac pro/antioxidant status in hypercholesterolemic rats [11, 12].

These numerous health benefits have led to an increasing demand for artichoke extract and have contributed to the spread of artichoke extract cultivation in many countries in Europe and beyond [13, 14].

The aim of this study was to evaluate the nutritional value of *Cynara scolymus* L. leaf extracts, with particular emphasis on the content and ratios of minerals, as well as the antioxidant activity (aa).

## Materials and Methods

### Plant Material

Extracts from leaves of the Green Globe variety artichoke (*Cynara scolymus* L.) were bought from Greenvit Botanical Extracts Manufacturer in Zambrów (52° 59′ 07″ N, 22° 14′ 35″ E, Poland). Dry extract (DER 2.5–7.5:1) was prepared in compliance with the assessment report EMA/HMPC/150218/2009 [15] on *C. scolymus* herb, which refers artichoke extracts for internal use.

### Basic Chemical Composition

Dry matter, crude protein, crude ash, and crude fat were determined according to procedures established by the Association of Official Analytical Chemists [16]. Total carbohydrates were calculated as total carbohydrates (%) = 100 − % (moisture + crude protein + crude fat + ash).

### Mineral Composition

The phosphorus content was determined by the Egner-Riehm colorimetric method, with ammonium molybdate, at 660 nm wavelength, on a Specol 221 apparatus. An Atomic Absorption Spectrometer apparatus (iCE 3000 Series, Thermo Fisher Scientific) was used to determine potassium, sodium, and calcium—by means of emulsion flame spectroscopy, and magnesium, zinc, iron, manganese, chromium, lead, cadmium, and nickel—by means of absorption flame spectroscopy. The material for macro-components concentration analyses was subjected to mineralization in concentrated sulfuric acid (H_2_SO_4_) and perchloric acid (HClO_4_). Material for micro-components concentration analyses was subjected to mineralization in a nitric acid (HNO_3_) and perchloric acid (HClO_4_) mixture.

### Determination of Total Phenolic Compound Content

The probes (dried) were used to prepare methanol extracts (2–3 g of probe in 80 ml of 70% methanol solution). In each case, dried samples of plant material were extracted by shaking (Elpan, water bath shaker type 357, Elpin-Plus, Lubawa Poland) at room temperature for 2 h, and solution was centrifuged (Centrifuge type MPW-340, MPW Medical Instruments, Warsaw, Poland), filtered and then the extracts were stored at *T* = − 22 °C. Methanolic extracts were used to measure the total phenolic compound content, using Folin-Ciocalteu reagent. This method involves colorimetric determination of colored products which are formed when polyphenolic compounds react with the Folin-Ciocalteu reagent (Sigma, St. Luis, MO, USA). The level of total polyphenolic compounds was determined spectrophotometrically (at a wavelength of *λ* = 760 nm using a RayLeigh UV-1800 spectrophotometer, Beijing Beifen-Ruili Analytical Instrument, Beijing, China) according to the Folin-Ciocalteu method [17]. Results were expressed as chlorogenic acid equivalents (CGA) in milligrams per 100 g of dry matter, based on a standard curve.

### Determination of Antioxidant Activity

#### ABTS˙^+^ Method

Methanol extracts were also used to determine antioxidant activity (aa) by measuring the sample’s ability to scavenge a free radical, i.e., ABTS˙^+^ (2, 2′-azinobis-(3-ethylbenzothiazoline-6-sulfonic acid) [18]. The absorbance was measured at a wavelength of *λ* = 734 nm using a RayLeigh UV-1800 spectrophotometer. Values obtained for each sample were compared to the concentration-response curve of the standard Trolox solution and expressed as micromoles of Trolox equivalent per 1 g of dry weight.

#### DPPH˙^+^ Method

The DPPH assay was done according to Miliauskas et al. [19] with some modifications. A stock solution of DPPH˙^+^ was prepared by dissolving 6 mg of 2,2-diphenyl-1-picrylhydrazyl in 100 ml of methanol. The working solution was obtained by diluting the stock solution with methanol to obtain an absorbance of 0.900–1.000 at 515 nm. Eighty microliters of the extract were transferred into a test tube and diluted with methanol to a total of 1.5 ml. After mixing of the diluted extract with 3 ml of the DPPH˙^+^ solution, the mixture was left in the dark at room temperature for 10 min. After this time, the absorbance was measured at 515 nm.

Values obtained for each sample were compared to the concentration-response curve of the standard Trolox solution and expressed as micromoles of Trolox equivalent per 1 g of dry weight.

### Ferric Reducing Power Method

The total reducing capability, according to the ferric reducing power (FRAP) method, was determined as reported previously by Benzie and Strain [20] with modifications. A working solution of FRAP reagent was prepared with 100 ml of 300 mM acetate buffer—pH 3.6, 10 ml of 10 mM 2,4,6-tris(2-pyridyl)-s-triazine (TPTZ) in a 40 mM hydrochloric acid solution, and 10 ml of 20 mM ferric chloride hexahydrate. A total of 10–200 μl of the extract was transferred into a test tube and diluted to 1 ml with 70% methanol. After mixing of the diluted extract with 3 ml of FRAP reagent working solution, the mixture was left in the dark at room temperature for 10 min. The absorbance of the samples was measured at 593 nm using a spectrophotometer (UV-1800, RayLeigh, Beijing Beifen-Ruili Analytical Instrument Co., Ltd., Beijing, China). Values obtained for each sample were compared to the concentration-response curve of the standard Trolox solution and expressed as micromoles of Trolox equivalent per 1 g of dry weight.

## Results and Discussion

In the available literature, there is almost no information on the basic composition of water extracts from artichoke leaves. In this study, the measured dry matter content was 95.4 g/100 g dry matter (DM) (Table 1) and protein content was 7.9 g/100 g DM. This is comparable to the findings of Magied et al. [21], who found 8.74 g protein/100 g DM in artichoke leaves. Our results are also similar to those of Hosseinzadeh et al. [22], who found a protein content (DM) in the range of 8.05 to 12.5%. Ruiz-Cano et al. [23] also showed 12.5 g crude protein in 100 g DM in different artichoke by-products from industrial canning processing.

**Table 1:** Chemical composition of *Cynara scolymus* L. leaves extract (g/100 g DM)

Specification	Content ± SD
Dry matter (% extract)	95.4 ± 0.1
Crude protein	7.9 ± 0.2
Crude fat	3.80 ± 0.04
Crude ash	13.400 ± 0.007
Total carbohydrates	75.0 ± 0.2

*DM* dry matter, *SD* standard deviations

The crude fat content of the artichoke leaf extract was 3.8 g/100 g DM. All artichoke by-product fractions assessed by Ruiz-Cano et al. [23] also contained low levels of fat (2.5–3.7% DM). Magied et al. [21] reported that the fat content of artichoke leaves in their study was only 2.34% DM. No crude fiber was found in the tested extract.

Carbohydrates made up the main component of the artichoke leaf extract. These included simple sugars, disaccharides, polysaccharides (starch), and some organic acids. The extract contained 75 g of total carbohydrates per 100 g DM, which was consistent with the results of other authors [23, 24].

The mean content of mineral compounds, determined as ash, was 13.4 g/100 g DM, confirming a high mineral content. Average concentrations of mineral components in the examined artichoke extract are presented in Table 2.

**Table 2:** Mineral composition of *Cynara scolymus* L. leaves extract (in 100 g DM)

Item	Content ± SD
Macroelements (mg)	
K	506.3 ± 1.5
P	414.0 ± 1.2
Ca	386.9 ± 13.0
Mg	220.7 ± 0.2
Na	194.4 ± 4.4
Microelements	
Zn (mg)	2.10 ± 0.03
Fe (mg)	1.60 ± 0.04
Mn (mg)	0.800 ± 0.005
Cr (μg)	1.500 ± 0.003
Pb, Cd, Ni	not detected

Levels of macroelement can be presented in descending order as follows: K > P > Ca > Mg > Na. Potassium (K) was the mineral present in the highest concentrations, with a mean level of 506.3 mg/100 g DM. The high K concentration in artichoke leaves has been documented before since K is the nutrient absorbed most by artichoke plants during their growing cycle [25]. According to Colla et al. [25], artichoke leaves have a phosphorus content of 890–980 mg/100 g DM, while our results showed a 40% lower level in the extract (414 mg/100 g DM).

According to our results, artichoke extract is also an important source of calcium (386.9 mg Ca/100 g DM) and magnesium. Magnesium content in the artichoke extract was 220.7 mg/100 g DM, which is consistent with the results of Orlovskaya et al. [26]. For comparison, a significantly lower amount of magnesium was found in gingko extract (*Ginkgo biloba* L.), another plant extract prized for its healthful properties and high content of minerals—0.3–0.7 mg/100 g DM [27].

In this study, artichoke extract contained 194.4 mg of sodium per 100 g DM. An analysis of sodium content in various medicinal raw materials performed by Arceusz et al. [28] showed that the average concentration of this element is very diverse, ranging from 3.4 mg/100 g DM to 75.8 mg/100 g DM. For example, the extract of the summer savory (*Satureja hortensis* L.) contains on average 8.4 mg Na/100 g DM [29], both significantly lower Na levels than in artichoke. Such a high sodium content in artichoke extract may contribute to an unhealthily high dietary sodium intake, which in Poland already often exceeds the adequate intake (AI) level. However, raw plant materials or extracts are usually used in small amounts and thus, their contribution to Na intake is minimal [30].

Trace elements such as zinc, manganese, copper, selenium, and iron play an important role in the fight against oxidative stress as components of enzymatic defense.

Zinc deficiency is often caused by malnutrition, consumption of poorly diversified foods, or disease conditions. In the studied artichoke extract, the zinc content was 2.1 mg/100 g DM. Similarly, Colla et al. [25] found zinc content in artichoke leaves in the range 2.07–2.35 mg/100 g DM. The Zn content in the artichoke extract in our analysis was similar to levels in the leaves of dandelion—1.91 mg/100 g DM [31].

The artichoke extract contained 1.6 mg of iron (Fe) per 100 g, but compared to birch leaves (16.5 mg/100 g DM) or raw mint leaves bag (23.9 mg/100 g DM), it is a very poor source of this mineral [31, 32]. The amount of chromium in the examined material was 1.5 μg/100 g DM.

The content of microelements in the artichoke extract could be presented in ranked as: Zn > Fe > Cr > Mn. Compared with the mineral composition of other vegetables reported in the literature, artichoke leaves are a good source of K and especially Zn, two of the most important essential elements in human nutrition [33].

With the current increase in the use and consumption of raw plant materials, it is very important to monitor contaminants present in plants and set maximum standards for their concentrations in order to ensure safe use. Lead and cadmium are the most toxic of the microelements, especially their inorganic compounds, which penetrate through mucous membranes into internal organs, especially to the liver, kidneys, and pancreas. No toxic elements (Pb, Cd, and Ni) were found in the tested extract.

The quality of 24-h food intake depends not only in the amount of individual minerals consumed but also in their proportions. Evaluation of these ratios is a better indicator of the nutritional quality of raw material than levels of individual elements. Table 3 shows the relations between elements in artichoke leaf extracts, calculated according to the recommended dietary allowance (RDA), adequate intake (AI), or tolerable upper intake level (UL) for men aged 31–50 years [34].

**Table 3:** Relations between elements *Cynara scolymus* L. leaves extract and calculated according to RDA or AI or UL

Elements ratios	Extract	Calculated according to RDA or AI or UL
Ca:P	0.93:1	1.43:1
Ca:Mg	1.75:1	2.38:1
K:Mg	2.29:1	11.19:1
Na:K	0.38:1	0,49:1
K:(Ca + Mg)	0.83:1	3.31:1
(K + Na): (Ca + Mg)	1.15:1	4.93:1
K:Ca	1.31:1	4.70:1
Fe:Mn	2.00:1	3.48:1
Mn:Zn	0.38:1	0.21:1
P:Zn	197.14:1	63.64:1

*RDA* recommended dietary allowance, *AI* adequate intake, *UL* upper intake level

Appropriate Ca:P ratio is an important factor for calcium-phosphate homeostasis. In the examined extract, the ratio of Ca:P was 0.93:1, which seems insufficient in comparison to the 1.43:1 recommended in Dietary Guidelines [34]. However, an even lower Ca:P ratio was found in buckwheat germ, where it ranged from 0.37:1 to 0.80:1 [35]. In the leaves of the common dandelion *Taraxacum officinale,* the Ca:P ratio was 1.31:1 [36], while in the leaves of the sea buckthorn *Hippophae rhamnoides,* it was only 0.61:1. In the hair of healthy women, the ratio ranged from 1.6 to 3.6:1 [37]. Of course, the quoted proportions in human hair cannot be directly compared, but analysis of the mineral composition of hair is an excellent tool in assessing the nutritional balance.

An appropriate level of magnesium in the diet is crucial for the transport of calcium and potassium through plasma membranes and of the transport of calcium to and from the bones [38]. In the tested artichoke extract, the Ca:Mg ratio was 1.75:1 and K:Mg ratio was 2.29:1, both significantly lower than those recommended by Dietary Guidelines [34]. In the leaves of the common dandelion *Taraxacum officinale*, the Ca:Mg ratio is 2.79:1, while in the leaves of sea buckthorn *Hippophae rhamnoides*, it is 1.14:1 [36]. The Ca:Mg ratio in hair is 3–11:1 [37].

Sodium and potassium, minerals critical for human metabolism, must also be in appropriate proportions in order to maintain the water-electrolyte balance in the body [39]. A prominent problem in the modern world is the excessive intake of sodium, for example, due to the consumption of highly processed food. Thus, consumers should try to enrich their diets with potassium to compensate for the high sodium levels and achieve a more desirable ratio. The Na:K ratio in the examined artichoke extract was 0.38:1, only slightly lower than that calculated in Dietary Guidelines [34]. Interestingly, Pongrac et al. [35] using natural mineral-rich water changed the ratio of these elements in buckwheat germs from 0.01:1 (control–tap water) to 175:1.

A small amount of manganese is absorbed from food through the gastrointestinal tract, which is influenced, among other things, by levels of iron and zinc. The iron-manganese ratio is additionally important as both minerals compete for the same serum protein (transferrin) and for the transport systems of the transport protein DMT-1 (divalent metal transporter) [40]. In our study, artichoke extract was characterized by an Fe:Mn ratio of 2:1 and a Mn:Zn ratio of 0.38:1. The former is lower, and the second higher than recommended, which suggests a slight relative excess of manganese in the extract. However, with a small intake of the extract, there is no risk of exceeding the highest tolerable intake level of some selected elements.

The elemental ratios determined for biomass (grain, straw) of plants intended for animal feed are highly variable [41–43] which limits the legitimacy of their comparison with the examined artichoke extract.

The antioxidant activity (aa) of plant raw materials can be determined by different methods. Many authors emphasize the necessity of performing more than one type of measurement in view of the multidirectional mechanisms of antioxidant compounds [44, 45]. Total phenolic content (TPC) in artichoke leaf extract was high and amounted on average to 2795 mg CAE (chlorogenic acid equivalents)/100 g DM (Table 4). Wang et al. [46] reported that the total phenolic content in Green Globe leaves was in the range of 8760 to 9561 mg CAE/100 g DM. Violet variety artichokes were characterized by lower total phenolic content in leaves, around 6800 mg CAE/100 g DM. Sałata and Gruszecki [47], in Green Globe artichoke leaves, estimated the total content of phenolic acids at 3167 mg caffeic acid equivalent/100 g DM. Phenolic compounds were dominated by chlorogenic acid, followed by cynarine and caffeic acid, which is notably different than in the methanol extracts studied by Wang et al. [46]. Kukić et al. [48] analyzed the total phenolic content in five extracts (including water extract) obtained from the involucral bracts of *C. cardunculus*. As much as 4600 mg CAE/100 g DM was measured in the water extract; however, the highest content of total polyphenols was found in *n-*BuOH extract and amounted to 6200 CAE/100 g DM (Table 4).

**Table 4:** Polyphenols content (TPC) and antioxidant activities (aa) of *Cynara scolymus* L. leaves extract

Item	TPC and aa
Polyphenols (mg CGA/100 g DM)	2795.0 ± 77.4
TEAC ABTS˙^+^ (μΜTrolox/1 g DM)	1060.8 ± 16.4
RSA_ABTS˙_ (%)	79.74 ± 0.66
TEAC DPPH˙^+^ (μΜTrolox/1 g DM)	261.9 ± 1.5
RSA_DPPH˙_ (%)	43.95 ± 0.08
TEAC TPTZ (FRAP) (μmolTrolox/1 g DM)	488.5 ± 2.5

*CGA* chlorogenic acid, *RSA* radical scavenging activity; *TEAC* Trolox equivalent antioxidant capacity

Gouveia and Castilho [49] found 233.6 mg GAE (gallic acid equivalent)/100 g DM in methanol artichoke leaf extract, which was significantly higher (by ca. 21%) than the level determined in *Madeira cardoon* leaf extract. Rhamos et al. [50], in methanol/water/acetic acid extract from artichoke leaves, found the presence of as much as 631 mg GAE/100 g DM. Ademoyegun et al. [51] analyzed the total phenolic content in the material from 25 leafy vegetables. The highest value observed was 164.52 mg GAE/100 g DM in methanol extract from *Sesamum radiatum* herb, which confirms the uniqueness of artichoke extract in this regard.

All of the above-mentioned phenolic content results were obtained using the Folin-Ciocalteu reagent. This method makes it possible to determine the content of all flavonoids, caffeic acid and its derivatives, and tannins. Meanwhile, methods based on HPLC also allow for the analysis of the profile of this group of compounds and indication of the resulting differences between species or varieties. Wang et al. [46] showed clear differences in the composition of phenolic compounds in the leaves of three artichoke varieties. The content of cynarine was similar, but the content of other compounds differed (1-O-caffeoylquinic acid, chlorogenic acid, luteolin rutinoside, and cynaroside).

Antioxidant activity of plant polyphenols is related to the presence and location of hydroxyl and methoxyl groups. Their presence facilitates the elimination of reactive oxygen species and chelation of metal ions [52]. The total content of phenols does not, however, allow precise determination of the ability to eliminate free radicals or the ability to reduce iron.

Measurements of antioxidant activity using several methods confirmed the high antioxidant properties of water artichoke leaf extract. The use of the ABTS˙^+^ radical proved a very high ability of artichoke extract to scavenge free radicals, as RSA was 79.74%, and the antioxidant capacity was measured as 1060.8 Trolox/1 g DM.

DPPH˙^+^ radical analysis showed a lower ability of artichoke extract to scavenge free radicals. The RSA in this case was nearly 44% and TEAC 261.9/1 g DM. Similar radical scavenging abilities of DPPH˙^+^ were demonstrated in the study by Wang et al. [46]. For the Green Globe variety in this study, RSA ranged from 40.5 to 49.7%. The significant difference in the evaluation of antioxidant activity using different radicals (ABTS˙^+^ vs. DPPH˙^+^) may be due to the fact that DPPH˙^+^ dissolves only in organic solvents and does not allow the determination of hydrophilic antioxidants. This is a specific limitation of this method, which thus does not give a full picture of the antioxidant properties of the tested extract. Such disagreement between antioxidant abilities determined by the aforementioned methods was also observed by Floegel et al. [53]. This difference was exacerbated in the case of heavily pigmented foods such as cherry, spinach, plum, and red cabbage. This is because ascorbic aside and phenolics are known to be hydrophilic antioxidants, while carotenoids are known to be lipophilic antioxidants [54].

The aqueous artichoke leaf extract also showed a significant ability to reduce iron (III) (TEAC = 466/1 g DM). Gouveia and Castilho [49] determined the antioxidant capacity of methanol artichoke leaf extract using the radicals ABTS˙^+^ and DPPH˙^+^ at 6.94 × 103 M Trolox/100 g and 3.77 × 103 M Trolox/100 g, respectively, which is significantly lower than shown in our study.

## Conclusion

The artichoke extract studied has a similar protein and fat content as that found in vegetable raw material and its composition is dominated by carbohydrates with a total absence of crude fiber. The ash content was 13.4%. The macroelement levels in the extract can be arranged in the following descending order: K > P > Ca > Mg > Na. The content of micronutrients decreased in the following manner: Zn > Fe > Cr > Mn. Some of the proportions determined for minerals differ significantly from those recommended by Dietary Guidelines, which should be considered when determining its dosage as a food additive. TPC was 2.8%, explaining the antioxidant properties of the tested extract. The ability to scavenge free radicals, depending on the method, ranged from 44 (DPPH˙^+^) to 80% (ABTS˙^+^). Due to its rich basic composition and high levels of antioxidants, *Cynara scolymus* leaf extract can likely be used in the prevention of chronic non-communicable diseases, namely those resulting from oxidative damage.
